# Estimating Individual Exposure to Malaria Using Local Prevalence of Malaria Infection in the Field

**DOI:** 10.1371/journal.pone.0032929

**Published:** 2012-03-29

**Authors:** Ally Olotu, Gregory Fegan, Juliana Wambua, George Nyangweso, Edna Ogada, Chris Drakeley, Kevin Marsh, Philip Bejon

**Affiliations:** 1 Kenya Medical Research Institute, Wellcome Trust Research Programme, Centre for Geographic Medicine Research, Kilifi, Kenya; 2 Nuffield Department of Medicine, Centre for Clinical Vaccinology and Tropical Medicine, University of Oxford, Oxford, United Kingdom; 3 London School of Hygiene & Tropical Medicine, London, United Kingdom; 4 Population Services International, Nairobi, Kenya; Walter & Eliza Hall Institute, Australia

## Abstract

**Background:**

Heterogeneity in malaria exposure complicates survival analyses of vaccine efficacy trials and confounds the association between immune correlates of protection and malaria infection in longitudinal studies. Analysis may be facilitated by taking into account the variability in individual exposure levels, but it is unclear how exposure can be estimated at an individual level.

**Method and Findings:**

We studied three cohorts (Chonyi, Junju and Ngerenya) in Kilifi District, Kenya to assess measures of malaria exposure. Prospective data were available on malaria episodes, geospatial coordinates, proximity to infected and uninfected individuals and residence in predefined malaria hotspots for 2,425 individuals. Antibody levels to the malaria antigens AMA1 and MSP1_142_ were available for 291 children from Junju. We calculated distance-weighted local prevalence of malaria infection within 1 km radius as a marker of individual's malaria exposure. We used multivariable modified Poisson regression model to assess the discriminatory power of these markers for malaria infection (i.e. asymptomatic parasitaemia or clinical malaria). The area under the receiver operating characteristic (ROC) curve was used to assess the discriminatory power of the models. Local malaria prevalence within 1 km radius and AMA1 and MSP1_142_ antibodies levels were independently associated with malaria infection. Weighted local malaria prevalence had an area under ROC curve of 0.72 (95%CI: 0.66–0.73), 0.71 (95%CI: 0.69–0.73) and 0.82 (95%CI: 0.80–0.83) among cohorts in Chonyi, Junju and Ngerenya respectively. In a small subset of children from Junju, a model incorporating weighted local malaria prevalence with AMA1 and MSP1_142_ antibody levels provided an AUC of 0.83 (95%CI: 0.79–0.88).

**Conclusion:**

We have proposed an approach to estimating the intensity of an individual's malaria exposure in the field. The weighted local malaria prevalence can be used as individual marker of malaria exposure in malaria vaccine trials and longitudinal studies of natural immunity to malaria.

## Introduction

Spatial heterogeneity in malaria exposure has been described at a micro-epidemiological level at varying transmission settings [1], [2]. It is responsible for variations in disease risk within a small area and is evidenced by geographical clustering of malaria infections. Approximately 80% of transmission occurs within 20% of the population [3], [4]. It has been attributed to factors such as varying ecologies of local malaria vectors[5], the pattern of contact between human host and vectors and intrinsic human host factors [6], [7].

Heterogeneity in malaria exposure may bias estimates of malaria vaccine efficacy over time in longitudinal studies [8], [9]. This is predicted by simulations of populations under heterogeneous malaria exposure, where vaccine efficacy is underestimated as a consequence of heterogeneity and apparent waning of efficacy over time is seen even if vaccine protection is maintained [10]. Although a randomized controlled trial may ensure equal distributions of malaria exposure at the start of the trial, if the vaccine is protective then the more highly susceptible individuals will experience earlier clinical malaria episodes in the control group than in the active vaccination group. Their subsequent removal from the “at risk set” will subsequently unsettle the comparability of vaccinees and non-vaccinees and produce inaccurate estimates of efficacy [8], [9]. This effect will become more marked as time since randomization increases. Furthermore vaccine efficacy may vary according to the intensity of exposure [11] and so estimating individual malaria exposure levels would allow an assessment of the interaction between vaccine effects and exposure.

Field studies investigating immunity to malaria face similar challenges to those encountered in vaccine trials. In such studies, groups of positive and negative individuals for a particular immunological variable at baseline are compared using relative risk estimate for an episode of malaria[12]. However, heterogeneity in malaria exposure makes it difficult to ascertain whether individuals who remain uninfected during the follow up have been exposed or not [13]. Inclusion of unexposed individuals in the analysis may result in a bias towards reduced estimates of immunity to malaria. Several approaches to circumvent this problem have been suggested. Individuals who develop neither a febrile episode nor asymptomatic parasitaemia during follow up might be considered unexposed. Exclusion of these unexposed individuals from the analysis strengthens the ascertainment of the effects of immunity, transmission intensity and age [14]. However the choice of individual exposure marker remains a challenge. Use of a positive blood film at a single time point may be inaccurate and could misclassify those whose parasitaemia had been cleared by anti-malaria drugs or immunity. Furthermore this approach does not take into account varied degrees of exposure levels. Some studies have used individual antibodies to schizont extracts as a marker of exposure [15], [16] or other recombinant malaria antigens[17]. This approach is validated as a marker of exposure at a population level [18], but at an individual level is complicated by variations in an individual's capacity to make antibodies to specific antigens and saturation effect of antibodies [13], [19].

Several statistical models have been proposed to adjust for heterogeneity of exposure [9], [20], but most are difficult to interpret since they are based on assumed distributions of malaria exposure within the population. It is not clear how to estimate an individual's level of exposure in the field. Entomological Inoculations Rates, parasite rates and infant conversion rates have frequently been used to describe exposure at the level of population, but are not readily applied to individuals.

The objective of this work was to examine alternative approaches to estimating individual exposure to malaria. We reasoned that the level of exposure to malaria can be inferred by proximity to other infected individuals at the local level. We therefore used data from three cohorts in Kilifi District to determine the relationship between the risk of malaria infection and measures such as; proximity to the next nearest infected and uninfected individual or the number of infected individuals in an area of a given radius. We also assessed the relationship between individual AMA1 and MSP1_142_ antibody levels and risk of malaria infection in a subset of children from one of the cohorts. We then determined the performance of these measures in correctly predicting cases of malaria infection.

## Methods

### Cohort population and data

We used cohort data from Chonyi, Junju and Ngerenya sub-locations located within Kilifi Health and Demographic Surveillance System (HDSS) [21]. The data were prospectively collected between 1999 and 2001 for Chonyi, 1998 and 2010 for Ngerenya and 2006 and 2010 for the Junju cohort.

Surveillance methods and detailed information on the cohorts have been previously published [22], [23]. In brief, participants were randomly selected from the study areas. Both weekly active surveillance by trained field workers and passive surveillance at health facilities were used to identify clinical malaria episodes. Blood smears were done in individuals with either a history of fever (For a Chonyi and Ngerenya cohorts only) or axillary temperature of 37.5 or more (All three cohorts). A cross sectional blood smear was done before long rains in all individuals regardless of the fever. In 291 children aged 5 to 17 months from the Junju cohort, a venous blood sample was obtained at a single cross sectional bleed and tested for anti-merozoite surface protein-1 (MSP-1_42_) and anti-apical membrane antigen-1 (AMA-1) human immunoglobulin (Ig) G antibodies by enzyme-linked immunosorbent assay as described previously [24]. Additional data collected included individual homestead locations (GIS coordinates).

For the purpose of this study, malaria infection was defined as any P. *falciparum* positive blood smears (i.e. either asymptomatic parasitaemia or an episode of febrile malaria). We also determined if each individual was living within a malaria hotspot [25]. Chonyi has been considered as a relatively high malaria transmission area with Junju and Ngerenya regarded as moderate and low malaria transmission areas respectively [26]. However since 1999 malaria has been declining in the overall study area [27].

### Assessing the relationship between malaria infection and proximity to infected case

We computed distances (in Kilometers) from each individual to all others in each of the cohort. The proximity of the index child to the next nearest infected child and next nearest uninfected child was calculated. This was done separately for two time windows; four months and one year time intervals. To derive the best powers for transforming distances, we fitted a set of power functions of distance as a function of malaria infection status in logistic regression models to optimize the log likelihood. This allowed for a nonlinear relationship to be fitted. The power functions that maximized the log likelihood fit were then used to transform absolute distances, and subsequently used in modified Poisson regression models to assess the effect of proximity to infected/uninfected children on the risk of malaria infection in the index child.

### Calculation of weighted local prevalence of malaria infection

The weighted local prevalence was calculated as distance-weighted proportions of malaria infected children within an area of specified radius and over specified time intervals. The time intervals used were four months and one year, in order to assess the temporal aspect of exposure. The four month interval reflected three distinct seasons with varying malaria transmission [28] whilst the one year time interval was selected as a convenient annual summary. We used inverse distance weighting to give the children nearest to the index more weight in determining the local prevalence [29].
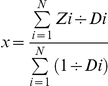



Where 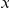 is the interpolated weighted local malaria prevalence for the index individual, 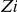 is the known infection status of the surrounding child (0: for uninfected and 1: for infected), 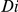 is the distance from the index individual to the surrounding child. The weighted local prevalence was expressed as a proportion with values between 0 and 1. We also calculated unweighted local malaria prevalence as the simple proportion of infected children within 1 km.

### Selection of best radius

To determine the best radius over which the weighted local prevalence should be calculated, we grouped children around each index child in the cohort into annuli at ≤0.2 km, >0.2 km to ≤0.5 km, >0.5 km to ≤1 km and >1 km to ≤2 km. We then determined how well the calculated weighted local prevalence from these annuli predicted the risk of individual malaria infection. The annuli analyses allowed us to determine if the individuals in the outer zones had any additional impact in the risk prediction. The cut-off point for the radius was based on the last distance beyond which the weighted local prevalence didn't predict risk of infection.

### Univariate analysis

The outcome measure was binary; malaria infection (i.e. either asymptomatic parasitaemia or at least one febrile malaria episode) or no malaria infection (i.e. no asymptomatic parasitaemia and no febrile malaria episodes) within four months or within one year time intervals. We investigated the effect of the following variables; weighted local malaria prevalence, distance to the next nearest infected and uninfected children and age. Residence in malaria hotspot as a binary variable was also included in the analysis because of prior report of its effect on risk of malaria infection [25]. A malaria hotspot was defined as an area where the observed incidence of febrile malaria or asymptomatic parasitaemia was higher than would be expected if cases were evenly distributed, as defined using the spatial scan statistic at p<0.05, including a maximum of 30% of the population in a hotspot.

In 291 children from Junju, the effect of log transformed AMA1 and MSP1 antibody levels on malaria infection were also assessed. The effect of each variable was assessed by modified Poisson regression analyses with a robust error variance [30].

Multiplicative interaction models were used to assess interactions between proximity to the infected and uninfected children on the risk of malaria infection in the index child. Adjustments were made for the multiple observations per individual with a fixed effect for the time period and random effect term for individual. Risk Ratios (RR) and 95% confidence intervals (95% CIs) were derived. To visualize the relationship between risk of malaria infection in the index child and proximity to other infected and uninfected children we differentiated the modified Poisson equation for the effect of distance and plotted the rate of change in risk over the first 1 km.

### Multivariable analysis and model calibration

A multivariable modified Poisson regression model was used to evaluate the independent role of each variable to predict malaria infection in the index child by including all significant variables (p<0.05) of univariate analysis.

We also used causal directed acyclic graph (DAG) as described before [31] to assess the suitability of our covariates for use in the final multivariable model. The aim was to minimize the magnitude of bias for the estimates of local malaria prevalence on the risk of malaria infection.

To evaluate the discriminatory ability of weighted local malaria prevalence and log transformed AMA1 and MSP1 antibody levels for malaria infection in the index child the area under the receiver operating characteristic (ROC) curve was determined [32]. The discriminatory power of individual models was compared with the model consisting of both anti-merozoite antibody levels and weighted local prevalence. Analyses were done using STATA version 11 software (Stata Corp., College Station, TX).

### Ethical consideration

Written informed consent was obtained from the adults enrolled and from parents/guardians of the young children enrolled using an approved consent form. The approval for human participation in three cohorts was given by the Kenya Medical Research Institute (KEMRI) National Ethical Review committee [23], [33].

## Results

A total of 2,425 participants were included in the final analysis constituting 7,166 person years of follow up. The age of participants ranged between 0 to 81 years (median 15, IQR; 0–76.2). There were 10,304 confirmed malaria infections of which 6,377 (62%) were asymptomatic. The demographic, parasitological characteristics and duration of follow-up for the three cohorts is shown in table 1.

**Table 1:** Demographic and parasitological characteristics of the cohorts used in the analysis.

Cohorts	Junju	Chonyi	Ngerenya
Follow up period	2006 to 2010	1999–2000	1998–2010
Age (median, IQR)	3.1(0.1–6.4)	15.6 (0.1–78.9)	14.5 (0–80)
Number of all participants (Percentage below 10 years)	620 (100)	874 (61.6)	931(66.9)
Female %	48.4%	58.7%	56%
Total number of malaria infections	2109	3283	4912
Asymptomatic infection	408	2480	3489
Total surveillance visits	83,566	90,437	200,074
Mode of surveillance	Active surveillance	Active surveillance	Active surveillance

### Risk of malaria as a function of proximity to the infected case

Increasing distance to the next nearest infected child was associated with a reduced risk of malaria infection in the index child in all three cohorts (RR = 0.37, 95%CI: 0.28–0.50 for Junju, RR = 0.18, 95%CI 0.03–0.84 for Chonyi and RR = 0.52, 95%CI 0.42–0.66 for Ngerenya). The rate of change in risk was highest within 1 km (Figure S1).

In contrast increasing distance to the next nearest uninfected child was associated with an increased risk of malaria infection in the index child; RR of 1.88 (95%CI: 1.30–2.72), 1.72 (95%CI: 1.48–2.0) and 1.49 (95%CI: 1.35–1.65) in Chonyi, Junju and Ngerenya respectively. The rate of change in risk was similarly highest within the first 1 km (Figure S1). We identified no interaction between the effects of distance to infected and uninfected children on the risk of malaria infection in the index child.

### Risk of malaria as a function of the weighted local malaria prevalence within a 1 km radius

The values of weighted local prevalence ranged between 0 and 1 and their distributions are shown in Figure S2. In Junju and Chonyi, weighted local malaria prevalence estimated from participants within ≤0.2 km, >0.2 km to ≤0.5 km, >0.5 km to ≤1 km but not those within >1 km to ≤2 km zones were predictive of malaria infection in the index child. In Ngerenya weighted local malaria prevalence estimated from participants within all four annulus were predictive of malaria infection in the index child (Table 2). We reasoned that because there was in inconsistent effect on the risk of malaria infection by the weighted local malaria prevalence beyond 1 km, but a consistent effect for the three zones examined within 1 km, that the optimal measure of exposure would be the distance-weighted proportion of malaria infections within 1 km radius. Consistently the plots of rate of change in risk of malaria infection versus proximity to infected case showed only a marginal effect beyond 1 km in all three cohorts (Figure S1).

**Table 2:** Effect of weighted local prevalence of malaria infection from four annuli around each individual on risk of malaria infection.

	RR(95%CI)	P value	AUC*
***Chonyi cohort***			
Weighted local malaria prevalence(<0.2 km)	2.19(1.78–2.70)	<0.001	0.68
Weighted local malaria prevalence (>0.2–0.5 km)	2.23 (1.66–3.02)	<0.001	0.68
Weighted local malaria prevalence (>0.5–1 km)	1.80 (1.279–2.55)	0.001	0.67
Weighted local malaria prevalence (>1–2 km)	1.49 (0.95–2.33)	0.079	NA
Unweighted local malaria prevalence<1 km	3.36 (1.66–6.77)	0.001	0.65
Weighted local malaria prevalence <1 km	2.19 (1.78–2.70)	<0.001	0.68
***Junju cohort***			
Weighted local malaria prevalence(<0.2 km)	1.95 (1.71–2.22)	<0.001	0.71
Weighted local malaria prevalence (>0.2–0.5 km)	1.42 (1.15–1.74)	0.001	0.68
Weighted local malaria prevalence (>0.5–1 km)	1.99 (1.22–3.24)	0.005	0.68
Weighted local malaria prevalence (>1–2 km)	0.71 (0.33–1.52)	0.383	NA
Unweighted local malaria prevalence<1 km	1.54 (1.18–2.01)	0.001	0.67
Weighted local malaria prevalence <1 km	1.99 (1.75–2.26)	<0.001	0.71
***Ngerenya cohort***			
Weighted local malaria prevalence(<0.2 km)	2.25 (1.90–2.67)	<0.001	0.82
Weighted local malaria prevalence (>0.2–0.5 km)	0.97 (0.69–1.36)	0.887	NA
Weighted local malaria prevalence (>0.5–1 km)	1.81 (1.44–2.27)	<0.001	0.81
Weighted local malaria prevalence (>1–2 km)	1.52 (1.13–2.04)	0.005	0.79
Unweighted local malaria prevalence<1 km	3.38 (2.58–4.42)	<0.001	0.80
Weighted local malaria prevalence <1 km	2.25 (1.90–2.67)	<0.001	0.82

* AUC: Area under the curve.

In a univariate analysis, weighted local malaria prevalence within 1 km was a strong predictor of risk of malaria infection in the index child in all three cohorts. An increase of 10% in weighted local malaria prevalence resulted in malaria infection RR of 1.99(95%CI: 1.75–2.26), 2.19(95%CI: 1.77–2.70) and 2.25 (95%CI: 1.90–2.67) in Junju, Chonyi and Ngerenya cohort respectively. Areas under the ROC curve for the univariate weighted local malaria prevalence models were 0.72(95%CI: 0.66–0.73), 0.71(95%CI: 0.69–0.73), and 0.82 (95%CI: 0.80–0.83) for Chonyi, Junju, and Ngerenya respectively.

The effect of unweighted local malaria prevalence was similar to weighted local prevalence with a tendency towards higher areas under ROC curve for distance-weighted than unweighted local malaria prevalence (Table 2).

We also examined the effects of weighted local malaria prevalence estimated from quarterly follow up data. These did not differ significantly from those estimated from yearly follow up data in Junju and Chonyi but was significantly higher in Ngerenya cohort (Table S1). The Areas under ROC curve were similar to those of yearly follow up in all the cohorts.

### Effect of malaria hotspot and age on the risk of malaria infection

Residence in a malaria hotspot was associated with an increased risk of malaria infection in the index child. The effect was more pronounced in the lowest transmission area; Ngerenya (RR: 1.45, 95%CI: 1.35–1.55) than in areas of moderate to high malaria transmission; Junju (RR; 1.29, 95%CI: 1.19–1.41) and Chonyi (RR: 1.23, 95%CI: 1.15–1.32) respectively. Age had a statistically significant non-linear effect on malaria infection in the index child. In all three cohorts risk of malaria infection increased with age and peaked at 5 years before starting a slow decline (Figure S3).

### Multivariable models for predicting risk of malaria infection

Multivariable models were separately developed for the three cohorts to assess the independent role of predictors of malaria infection in the index child and to determine the overall discrimination achieved with the multivariable model. The final multivariable model incorporated the weighted local malaria prevalence within a 1 km radius, distance to the next nearest infected child, distance to the next nearest uninfected child, age and whether resident in a malaria hotspot. Using DAG approach we confirmed that all selected covariates were plausible confounders and their inclusion in the final model would minimize the magnitude of the bias in the estimate of effect of local malaria prevalence on the risk of malaria infection (Figure S4).

**Figure 1:**
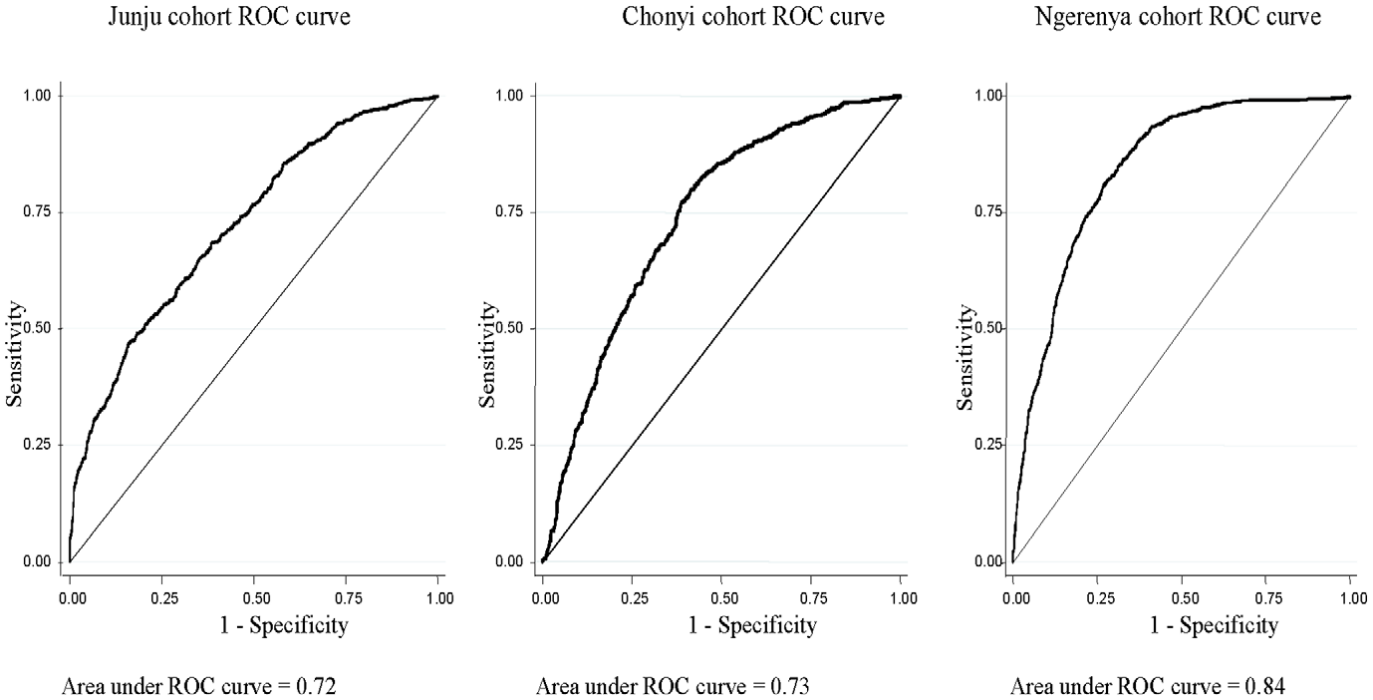
Areas under the ROC curves for the Multivariable weighted local prevalence based models for the three cohorts.

Weighted local malaria prevalence, location within a malaria hotspot and age remained significant predictors of malaria infection in the multivariable model (Table 3). Proximity to the nearest infected child was predictive in Chonyi but not in Junju and Ngerenya. The areas under the ROC curve for the multivariable prediction models were 0.74 (95%CI: 0.72–0.76) 0.72 (95%CI: 0.70–0.74), and 0.84 (95%CI: 0.83–0.85) for the Chonyi, Junju, and Ngerenya cohorts, respectively (Figure 1).

**Table 3:** Univariate and Multivariable analysis of predictors of malaria infections.

	Univariate analysis		Multivariable analysis	
	RR(95% CI	P value	RR (95% CI	P value
***Chonyi cohort***				
Weighted local prevalence (1 km radius)§	2.19(1.77–2.70)	<0.001	1.78(1.38–2.29)	<0.001
Proximity to nearest infected case§	0.18 (0.03–0.84)	0.03	0.33(0.07–1.40)	0.135
Proximity to the second nearest case§	1.07(0.65–1.76)	0.763	NA#	
Proximity to the nearest uninfected case§	1.88(1.30–2.72)	0.001	1.13(0.78–1.65)	0.499
Proximity to the second nearest uninfected case§	0.93(0.72–1.21)	0.619	NA#	-
Residence in malaria hotspot	1.23(1.15–1.32)	<0.001	1.14(1.06–1.22)	<0.001
Age (years)*	NA	<0.001	NA	<0.001
***Junju cohort***				
Weighted local prevalence (1 km radius)§	1.99(1.75–2.26)	<0.001	1.51(1.21–1.87)	<0.001
Proximity to nearest infected case§	0.37(0.28–0.50)	<0.001	0.57(0.40–0.81)	0.002
Proximity to the second nearest case§	0.74(0.62–0.87)	<0.001	NA#	-
Proximity to the nearest uninfected case§	1.72(1.48–2.0)	<0.001	1.16(0.93–1.43)	0.172
Proximity to the second nearest uninfected case§	1.63(1.40–1.90)	<0.001	NA#	-
Residence in malaria hotspot	1.29(1.19–1.41)	<0.001	1.19(1.10–1.30)	<0.001
Age (years)*	NA	<0.001	NA	<0.001
***Ngerenya cohort***				
Weighted local prevalence (1 km radius)§	2.25 (1.90–2.67)	<0.001	1.49(1.24–1.81)	<0.001
Proximity to nearest infected case§	0.52(0.42–0.66)	<0.001	0.52(0.38–0.71)	<0.001
Proximity to the second nearest case§	0.77(0.56–1.05)	0.101	NA#	
Proximity to the nearest uninfected case§	1.49(1.35–1.65)	<0.001	1.07(0.93–1.24)	0.286
Proximity to the second nearest uninfected case§	1.42(1.31–1.54)	<0.001	1.17(1.05–1.30)	0.003
Residence in malaria hotspot	1.45(1.35–1.55)	<0.001	1.26(1.16–1.36)	<0.001
Age (years)*	NA	<0.001	NA	<0.001

*Multivariable polynomial fraction showed age has a non linear effect in all the cohorts (see Figure S3),

# : The best fit model was obtained with only first nearest distances in the model,

§ : Risk ratios are for each step increase in 0.35 and 0.45 power function of distance to the infected and uninfected child respectively.

### Local malaria prevalence and Merozoite antibodies based models in predicting malaria risk

Merozoite antibody levels were assessed in 291 children in the Junju cohort (median age 20.5 months, IQR: 11.6–28.1) at a cross sectional bleed. Merozoite antibodies levels were associated with increase in prospective risk of malaria infection in the index child (Table 4). Univariate predictive models for AMA-1 and MSP-1_42_ antibodies produced areas under the ROC curve of 0.75 and 0.76 respectively.

**Table 4:** Merozoite antibody versus weighted local prevalence based models in predicting malaria infection in a Junju sub-cohort.

	RR	[95% CI]	P value	AUC* (95%CI)
***Univariate specific antibody-based model***				
AMA1	2.27	1.80–2.86	<0.001	0.75 (0.70–0.80)
MSP1_142_	2.03	1.70–2.42	<0.001	0.76 (0.72–0.82)
***Multivariable weighted local prevalence-based model***				
Weighted local prevalence	2.29	1.22–4.30	0.009	0.72 (0.67–0.76)
Malaria hotspot	1.16	0.89–1.51	0.245	
Proximity to the nearest infected case	0.76	0.43–1.32	0.337	
Proximity to the nearest uninfected case	1.08	0.62–1.87	0.768	
***Univariate weighted local prevalence-based model***				
Weighted local prevalence	3.00	2.28–3.94	<0.001	0.69 (0.64–0.73)
***Combined weighted local prevalence and anti-merozoite antibody***				
Weighted local prevalence (1 km)	2.14	(1.60–2.87)	<0.001	0.83(0.79–0.88)
AMA1	1.36	(1.06–1.74)	0.015	
MSP1_142_	1.56	(1.28–1.89)	<0.001	

*AUC: Area under the curve.

In the same group of children weighted local malaria prevalence within 1 km radius was associated with the risk of malaria in the index child in the univariate model providing area under ROC curve of 0.69 (95%CI: 0.64–0.73). A multivariable model incorporating weighted local malaria prevalence, distance to the next nearest infected, distance to the next nearest uninfected children and residence in a malaria hotspot had an area under the ROC curve of 0.72 (95%CI: 0.67–0.76) which was not markedly different from either weighted local malaria prevalence or antibody level specific univariate models. The area under the ROC curve for the multivariable model incorporating weighted local malaria prevalence and antibodies to AMA1 and MSP_142_ was 0.83 (95%CI: 0.79–0.88) (Table 4).

## Discussion

Being able to quantify an individual's malaria exposure in the field will allow a more precise analysis of the efficacy of candidate malaria vaccines in clinical trials, and of the potential immune correlates associated with protection from malaria. Based on this study we propose a measure of individual malaria exposure that uses the distance-weighted local prevalence of malaria infection (composite endpoint including asymptomatic infection or febrile malaria) within a 1 km radius. The measure is empirical, being derived from active malaria surveillance and location data, and not based on any assumed distribution of exposure. The weighted local malaria prevalence demonstrated moderate to good discriminatory ability for malaria infection in the index child (ROC of 0.71, 0.72 and 0.82 in Junju, Chonyi and Ngerenya respectively). The discriminatory ability of a multivariable model incorporating the distance-weighted local malaria prevalence (within a 1 km radius), age, distance to the next nearest infected, distance to the next nearest uninfected children and the presence or absence of a malaria hotspot was not statistically different from that of distance-weighted local prevalence within a 1 km radius alone (Table 4).

In 291 children in Junju who had antibody levels measured, merozoite surface protein-1 (MSP-1_142_) and apical membrane antigen-1 (AMA-1) antibody levels were also good predictors of the individual prospective risk of malaria infection as described before [24], [34] and their discriminatory ability for malaria infection was comparable to that of weighted local malaria prevalence. The combined model incorporating both of the antibodies data as well as and the weighted local malaria prevalence had slightly higher discriminatory ability than either alone (ROC of 0.83). Weighted local malaria prevalence captures exposure related to the spatial distribution of local infections. However antibody responses likely reflects both geographical variations in exposure and individual variations resulting from factors such as bed net use, individual attractiveness to mosquitoes [6] or genetic variation in susceptibility [35]. This could explain the improved predictive power of the model incorporating the two measures. However, using antibody levels as marker of exposure could be circular in observational studies of natural immunity, particularly when one intends to assess the potential protective value of same antibody response or a closely correlated antibody response. Under such circumstances adjusting for weighted local malaria prevalence as a marker of exposure may improve the estimates of antibody effect. Furthermore, antibody levels to blood stage antigens may be misleading if half the cohort has been randomized to a pre-erythrocytic vaccine that prevents exposure to blood stage parasites. On the other hand, provided a standardized assay is used, antibody levels will be more readily generalized between cohorts, and give an indication of the average transmission intensity of the cohort that can be compared with other cohorts.

Heterogeneous exposure to malaria complicates the analysis of efficacy of candidate malaria vaccines [10]. Calculating the weighted local prevalence of malaria infection for each child will allow for more sophisticated analyses, such as dividing the cohort into “high exposure” and “low exposure” groups, and examining interactions between intensity of malaria exposure and vaccination. Other indirect measures of exposure such as entomological inoculation rate and parasite prevalence may also be used at a larger scale in large multi-centre study involving sites with known transmission intensities. However for a single site such measures will provide only the average exposure for the population and not reflect the underlying variability of exposure at homestead or individual level.

To avoid circular reasoning we avoided using index child's own malaria infection status to calculate the individual weighted local malaria prevalence. Our causal diagram proposed additional cause for malaria hotspot comprising of unmeasured environmental factors. Therefore, although both local malaria prevalence and malaria hotspot shared spatial transmission factors as common ancestor, they represented two different causal pathways to sporozoites exposure. This could explain why the effect of malaria hotspot and local malaria prevalence remained significant in the multivariable model.

The risk of malaria infection (i.e. the composite endpoint of asymptomatic and symptomatic parasitaemia) increased with age early on in life and decreased with age later in life consistent with findings from previous studies [36]. Lower exposure to mosquito bites due to small body surface area in children could explain the early trend [37], and the apparent observed decline in the risk of malaria infection later in life could be attributed to the development of effective pre-erythrocytic immunity or of blood stage immunity which suppresses asymptomatic parasitaemia below the level of detection [38].

Our study has limitations. Our surveillance approach identifies acute clinical malaria by weekly surveillance and asymptomatic parasitaemia on yearly cross-sectional blood films. We would therefore miss brief asymptomatic infections, asymptomatic infections below the level of detection by microscopy, and exposure that does not result in a blood stage infection because of pre-erythrocytic immunity. Nevertheless we have identified here empiric evidence that weighted local malaria prevalence predicts the risk of malaria infection in the index child with reasonable accuracy. We infer that the bias resulting from the limitations described do not preclude the utility of the approach. Furthermore, these limitations may result in an under-estimate of the local prevalence of infection, but in the absence of a geographical bias, the local prevalence will still reflect the intensity of exposure relative to the rest of the cohort.

Our findings may not be directly applicable to other settings where the transmitting vectors and human behavior patterns vary. The optimal radius for calculating local prevalence may be different, and the relative predictive power of malaria hotspots, weighted local malaria prevalence and antibody levels would reflect the local setting. However, heterogeneity on a fine-scale is observed in many different settings [2], [39], [40] and it is likely that our approach to determining weighted local malaria prevalence could be adapted to these settings given adequate data.

We have assumed that individuals remained in the same location. Although most infections are likely to be acquired in the evening or night when individuals are at the homestead, it is possible that some infections were acquired during travel and this is not captured in the calculation of the weighted local malaria prevalence. Finally the described analysis was possible given the existence of continuous population based surveillance in Kilifi, something which may not be applicable in other settings.

In conclusion we have used a conceptually straightforward approach to generate weighted local malaria prevalence as an estimate of individual's intensity of exposure to malaria. We have demonstrated that the weighted local malaria prevalence has satisfactory discriminatory ability, particularly when combined with anti-merozoite antibody levels. We propose that it could be used as general marker of exposure to malaria and used as a covariate in models assessing the efficacy of potential malaria vaccines or immune correlates of protection to adjust for the heterogeneity in malaria exposure.

## Supporting Information

Figure S1 **Rate of change in risk of malaria infection as a function of distance to infected and uninfected in the first Kilometer.** Y axis represents a change in risk coefficient per unit increase in kilometer from infected or uninfected case.
(TIF)

Figure S2 **Distribution of weighted local prevalence of malaria in the three cohorts by year of follow up.** * No case of malaria infection was identified in the Ngerenya cohort between 2008 and 2010.
(TIF)

Figure S3 **Multivariable Fractional polynomial plots of effect of age on the risk of malaria infection in three cohorts.**
(TIF)

Figure S4 **Causal directed acyclic graphs (DAG).** Panel A represents the causal diagram for the data Panel B represent causal diagram after 6 step DAG approach and if one conditions on Age, malaria hotspot, spatial transmission factors (distance from infected and uninfected children) and blood stage antibodies (dashed boxes). Dotted lines represent conditional associations.
(TIFF)

Table S1 **Weighted local prevalence of malaria infection for four monthly follow-up data.** Multivariable polynomial fraction showed age has a non linear effect in all the cohorts (see Figure S3).
(DOC)
