# Supplementary figures and images for: Estimating Individual Exposure to Malaria Using Local Prevalence of Malaria Infection in the Field
Source: PLoS One. 2012 Mar 29;7(3):e32929. doi: 10.1371/journal.pone.0032929 (PMC3315550; doi:10.1371/journal.pone.0032929)

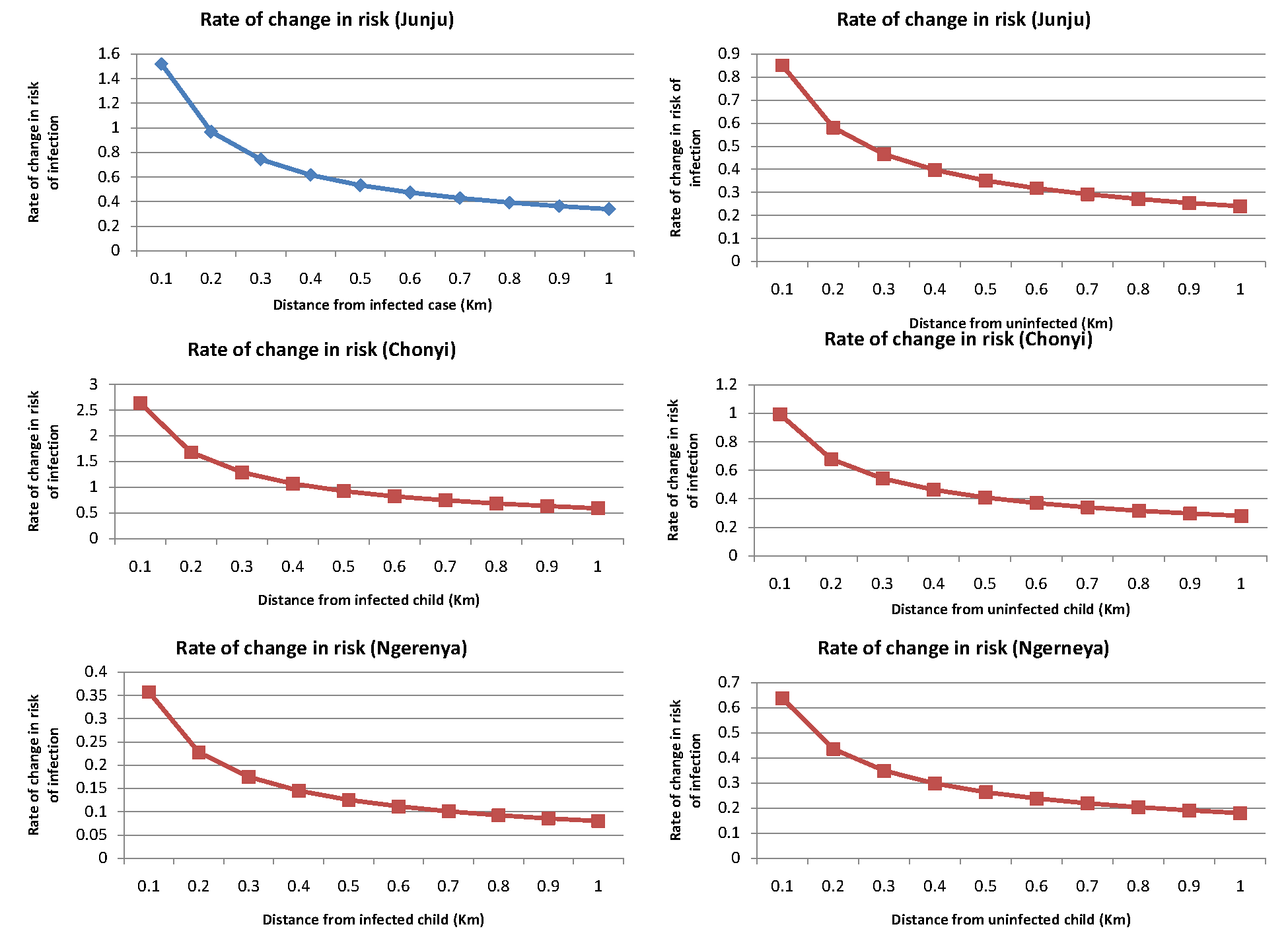

Supplement: Figure S1 — Rate of change in risk of malaria infection as a function of distance to infected and uninfected in the first Kilometer. Y axis represents a change in risk coefficient per unit increase in kilometer from infected or uninfected case. (TIF) [file pone.0032929.s001.tif]

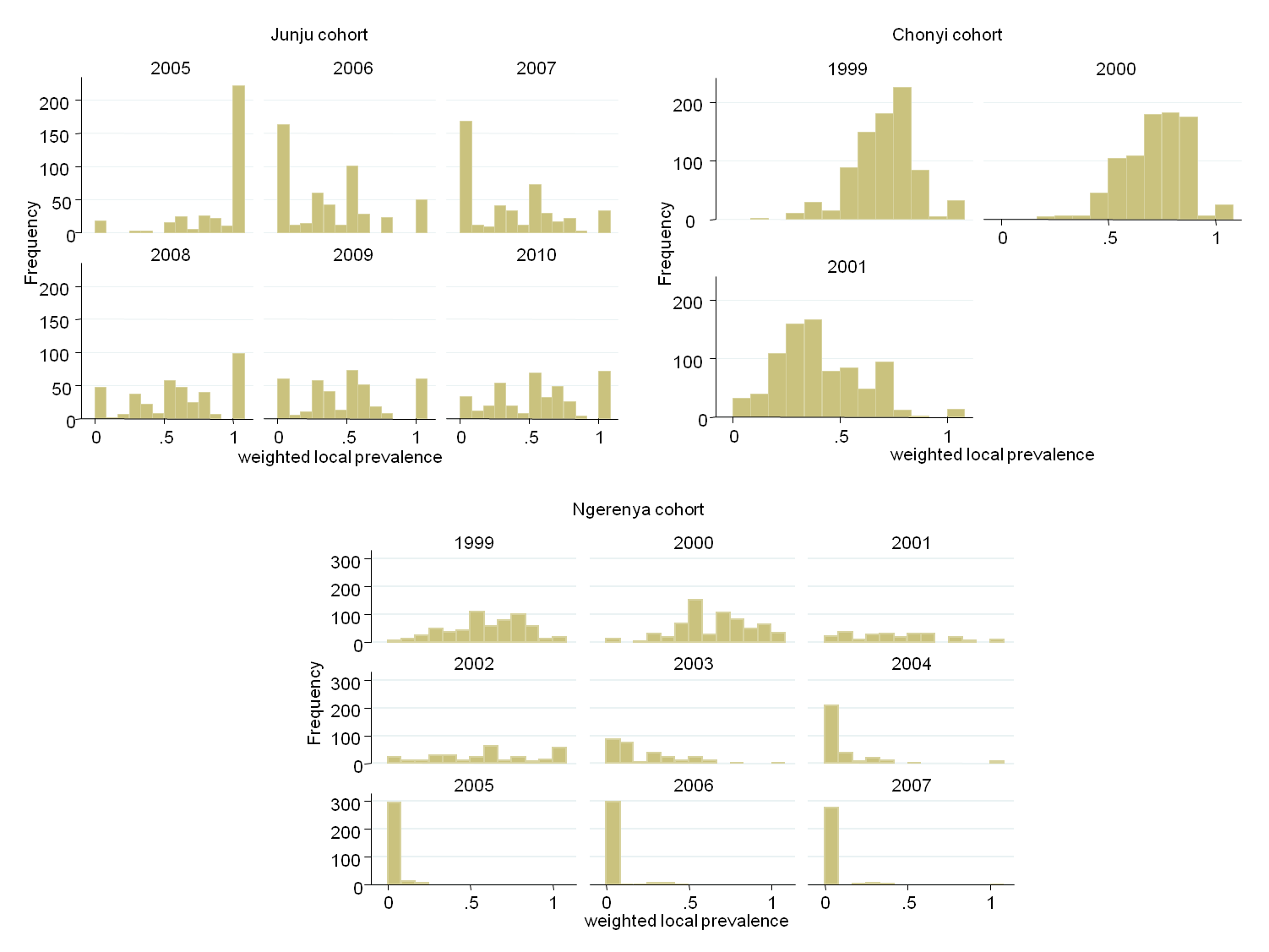

Supplement: Figure S2 — Distribution of weighted local prevalence of malaria in the three cohorts by year of follow up. * No case of malaria infection was identified in the Ngerenya cohort between 2008 and 2010. (TIF) [file pone.0032929.s002.tif]

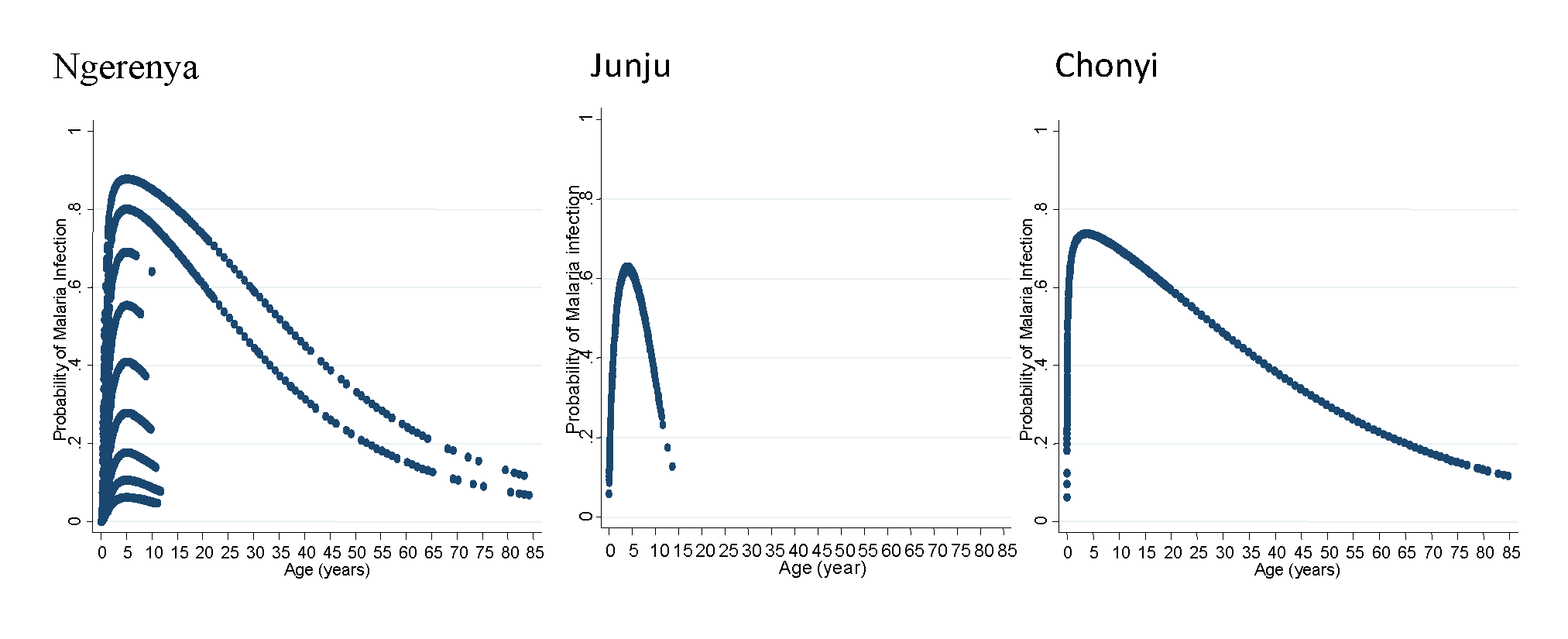

Supplement: Figure S3 — Multivariable Fractional polynomial plots of effect of age on the risk of malaria infection in three cohorts. (TIF) [file pone.0032929.s003.tif]

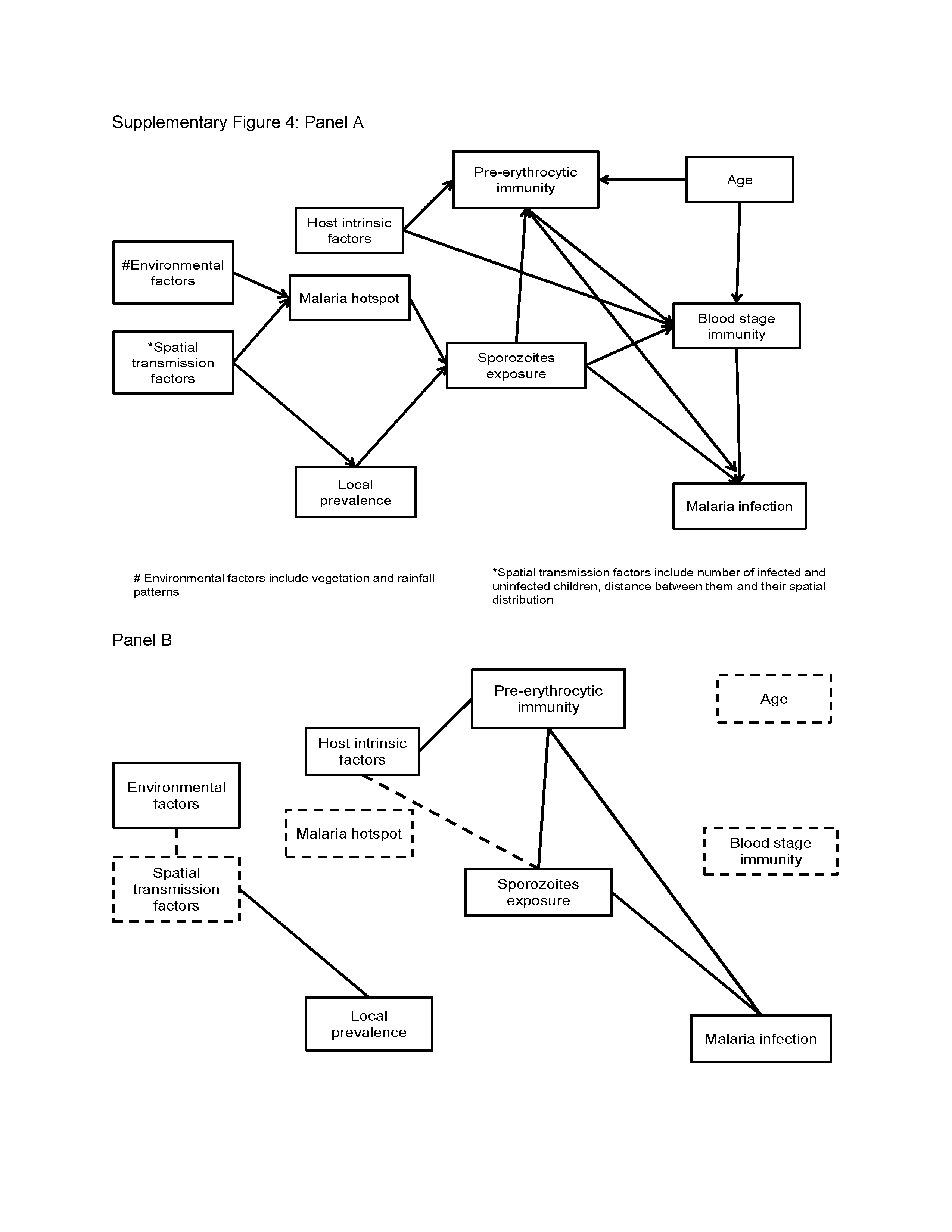

Supplement: Figure S4 — Causal directed acyclic graphs (DAG). Panel A represents the causal diagram for the data Panel B represent causal diagram after 6 step DAG approach and if one conditions on Age, malaria hotspot, spatial transmission factors (distance from infected and uninfected children) and blood stage antibodies (dashed boxes). Dotted lines represent conditional associations. (TIFF) [file pone.0032929.s004.tif]
