# Supplementary material for: Estimating Individual Exposure to Malaria Using Local Prevalence of Malaria Infection in the Field
Source: PLoS One. 2012 Mar 29;7(3):e32929. doi: 10.1371/journal.pone.0032929 (PMC3315550; doi:10.1371/journal.pone.0032929)
Supplement: Table S1 — Weighted local prevalence of malaria infection for four monthly follow-up data. Multivariable polynomial fraction showed age has a non linear effect in all the cohorts (see Figure S3). (DOC) [file pone.0032929.s005.doc]

**Supplementary Table 1: Weighted local prevalence of malaria infection for four monthly follow-up data**

|  | **Univariate analysis** | | **Multivariable analysis** | |
| --- | --- | --- | --- | --- |
|  | RR (95%CI) | P value | RR (95%CI) | P value |
| ***Ngerenya cohort*** | | | | |
| Weighted mean incidence (radius 1km) | 4.55(3.76- 5.51) | <0.001 | 2.59(2.11-3.18) | <0.001 |
| Distance to nearest infected case | 0.46(0.38-0.54) | <0.001 | 0.73(0.6-0.90) | 0.003 |
| Distance to the nearest uninfected case | 2.19(1.91-2.53) | <0.001 | 1.17(0.98- 1.41) | 0.080 |
| Residence in malaria hotspot | 1.89(1.74 -2.06) | <0.001 | 1.58(1.43-1.73) | <0.001 |
| Age* | NA | <0.001 | NA | <0.001 |
| ***Chonyi cohort*** | | | | |
| Weighted mean incidence (radius 1km) | 2.48(2.06- 2.98) | <0.001 | 1.55(1.31-1.84) | <0.001 |
| Distance to nearest infected case | 0.20(0.11- 0.38) | <0.001 | 0.3(0.16- 0.57) | <0.001 |
| Distance to the nearest uninfected case | 2.54(1.77- 3.61) | <0.001 | 1.68(1.31- 2.14) | <0.001 |
| Residence in malaria hotspot | 1.47(1.36- 1.59) | <0.001 | 1.34(1.25-1.44) | <0.001 |
| Age* | NA | <0.001 | NA | <0.001 |
| ***Junju cohort*** | | | | |
| Weighted mean incidence (radius 1km) | 2.09(1.84- 2.36) | <0.001 | 1.70(1.32- 2.18 | <0.001 |
| Distance to nearest infected case | 0.43(0.35-0.52) | <0.001 | 0.66(0.49-0.89) | 0.007 |
| Distance to the nearest uninfected case | 1.5(1.21-1.86) | <0.001 | 0.86(0.61-1.20) | 0.369 |
| Residence in malaria hotspot | 1.37(1.25-1.51) | <0.001 | 1.29(1.18-1.41) | <0.001 |
| Age* | NA | 0.37 | NA | 0.39 |

*Multivariable polynomial fraction showed age has a non linear effect in all the cohorts (see Figure S3)
